# Neighbourhood immigrant concentration effects on migrant and native youth’s educational commitments, an enquiry into personality differences

**DOI:** 10.1177/0042098016640693

**Published:** 2016-03-31

**Authors:** Jaap Nieuwenhuis, Pieter Hooimeijer, Maarten van Ham, Wim Meeus

**Affiliations:** Delft University of Technology, Netherlands; Utrecht University, Netherlands; Delft University of Technology, Netherlands; University of St Andrews, UK; Utrecht University, Netherlands; Tilburg University, Netherlands

**Keywords:** adolescents, educational commitment, migrant youth, neighbourhood effects, personality

## Abstract

In the literature examining neighbourhood effects on educational outcomes, the socialisation mechanism is usually investigated by looking at the association between neighbourhood characteristics and educational attainment. The step in between, that adolescents actually internalise educational norms held by residents, is often assumed. We attempt to fill this gap by looking at how the internalisation of educational norms (commitments) is influenced by neighbourhoods’ immigrant concentration. We investigate this process for both migrant and native youth, as both groups might be influenced differently by immigrant concentrations. To test our hypothesis we used longitudinal panel data with five waves (N = 4255), combined with between-within models which control for a large portion of potential selection bias. These models have an advantage over naïve OLS models in that they predict the effect of change in neighbourhood characteristics on change in educational commitment, and therefore offer a more dynamic approach to modelling neighbourhood effects. Our results show that living in neighbourhoods with higher proportions of immigrants increases the educational commitments of migrant youth compared to living in neighbourhoods with lower proportions. Besides, we find that adolescents with a resilient personality experience less influence of the neighbourhood context on educational commitments than do adolescents with non-resilient personalities.

## 摘要

在关于教育成果的邻里效应的研究文献中，社会化机制的研究方式通常是考察邻里特征与教育获得度之间的关联。这种方式往往预设了中间的一道逻辑：青少年实际上内化了居民的教育准则。我们尝试通过考察邻里的移民聚集度如何影响教育准则（投入度）的内化，来填补这道缺口。我们针对移民青年和本地青年分别考察了这一进程，因为这两个群体可能在不同程度上受到移民聚集度的影响。为验证我们的设论，我们采用了带五个波段的纵向面板数据 (N = 4255)，并结合组间-组内模型，把大量潜在选择偏差作为控制变量。这些模型相比传统最小平方法 (naïve OLS) 模型的优点是预测了邻里特征的变化对教育投入度变化的影响，因此提供了对邻里效应建模的更动态方式。我们的结果显示，居住在移民比例更高的邻里社区，相比居住在移民比例较低的邻里社区，移民青年的教育投入度更高。此外，我们发现，性格富有弹性的青少年，相比性格没有弹性的青少年，所经历的邻里环境对教育投入度的影响要低。

## Introduction

Many studies have tried to link adolescents’ educational achievement to the quality of the neighbourhood environment in which they grow up (so-called neighbourhood effects; for an overview of the neighbourhood effects literature see: Dietz, 2002; Ellen and Turner, 1997; Galster, 2002; van Ham et al., 2012). There is substantial debate with little apparent agreement on the causal mechanisms which produce neighbourhood effects, and their relative importance in shaping individuals’ life chances compared to other influences (van Ham et al., 2012). One mechanism states that the neighbourhood context might influence educational outcomes through processes of socialisation, where neighbourhood residents hold certain norms and transmit these norms to other residents. A high presence of positive attitudes towards education in neighbourhoods can be expected to reflect positively on adolescents. Adolescents are more inclined to adopt positive attitudes when they have positive role models showing the merits of education (Ainsworth, 2002; Wilson, 1996), and when there is more adult interference and social control in cases such as truancy (Akers et al., 1979; Galster, 2011; Sampson and Raudenbush, 1999). The neighbourhood effects thesis suggests that because of the presence of certain norms in the neighbourhood, adolescents will internalise these norms (educational commitments), which consequently has an effect on their educational achievement.

Most studies have only looked at the relationship between the neighbourhood context and educational outcomes, including final grades, graduation and highest achieved education (for reviews, see: Leventhal and Brooks-Gunn, 2000; Nieuwenhuis and Hooimeijer, 2015). In this paper, however, we will focus on the first link, i.e. the relationship between the neighbourhood context and whether adolescents internalise certain educational norms. More specifically, we will look at how the neighbourhoods’ immigrant concentrations affect the educational commitments of migrant and native adolescents. We focus on immigrant concentrations because we expect that, for youth with an immigrant background, the above mentioned socialisation processes may be most dependent on neighbourhood levels of immigrant concentrations, since individuals tend to be focussed, in terms of social relationships, on their own group (McPherson et al., 2001).

The concept of educational commitment has shown to be positively related to better school performance (Germeijs and Verschueren, 2007; Robbins et al., 2004), and is positively associated with the ability to adjust to the educational demands of the university (Luyckx et al., 2006), scholastic competences, work ethic and achievement motivation (Meeus et al., 2002). When we refer to educational commitment, we refer specifically to ‘identification with educational commitments’, which is distinctive from ‘making educational commitments’. Making educational commitments refers to the degree to which adolescents have made choices in the educational domain, and are committed to these choices. However, making a commitment is not the same as identifying with and feeling certain about that commitment. This is captured in the concept identification with educational commitments, which refers to the degree that adolescents identify themselves with, feel certain about and internalise the educational commitments they made (Luyckx et al., 2006; for a review, see Meeus, 2011). The strength of an adolescent’s educational commitments indicates the goals and values that an adolescent has set for his/her life. This definition of educational commitments makes it an ideal candidate to test how neighbourhood characteristics influence the internalisation of certain educational values. By comparing migrant youth with native adolescents, we attempt to find out whether there are differences in how the neighbourhood context influences the educational commitments of both groups and why some youths do better in education than others.

When looking at neighbourhood effects, we should keep in mind that different individuals within neighbourhoods might be affected differently. This relates to one of the main problems in the neighbourhood effects literature, that the outcomes of empirical studies into neighbourhood effects are biased by unmeasured characteristics of individuals: unobserved heterogeneity in research samples obscures ‘true’ neighbourhood effects. In this paper we argue that personality might be such a commonly unobserved individual trait that can affect the measured outcomes of neighbourhood effect studies. In previous studies on educational achievement, unemployment and work commitment we found that adolescents with a resilient personality are better able to cope with neighbourhood adversity, and therefore less likely to be affected by neighbourhood characteristics than adolescents with a less resilient personality (Nieuwenhuis et al., 2015a, 2015b). In the current study we will investigate to what extent adolescents with different personalities experience a different effect of the neighbourhood on their educational commitments. We hypothesise that adolescents with a resilient personality are better able to cope with neighbourhood stressors, and will therefore experience less influence of neighbourhood characteristics on their educational commitment than adolescents with non-resilient personalities.

We want to note that selection bias is a problem for most neighbourhood research. Neighbourhoods are not random selections of households, but families sort into neighbourhoods according to their preferences and economic constraints. When studies do not properly control for this problem, found neighbourhood effects could be overestimated or underestimated. Therefore, to test our hypothesis we used longitudinal panel data consisting of five waves (N = 4255), which enables us to use between-within models, which take care of time-invariant unobserved variables that have the potential to cause selection bias (Allison, 2009).

## Literature review

Based on a literature study we will first develop a hypothesis for the effect of the degree of immigrant concentration in neighbourhoods on youth’s educational commitments. Next, we will elaborate on how having a resilient personality may alter the relationship between neighbourhoods’ immigrant concentration and educational commitment.

### Immigrant concentration

A high proportion of non-western immigrants in a neighbourhood is often considered as undesirable by local and national governments because it can hinder the integration of immigrants in the host society. It can be argued that, for residents with an immigration background, contacts with natives are more beneficial than contacts with co-ethnics, since natives have in general better knowledge about, for example, jobs and the educational system, and therefore can provide access to the host society (Burt, 2001; Putnam, 2000). People’s social networks are often influenced by the composition of their environment (Mollenhorst et al., 2008). It was found that, in the Netherlands, immigrants living in neighbourhoods with a higher proportion of immigrants have a lower likelihood to include Dutch people in their social network (Martinovic et al., 2009). A high proportion of immigrants in the neighbourhood is to some extent a proxy for neighbourhood disadvantage, since immigrants are lower educated, have more unemployment, earn less and are less satisfied with their living environment compared to native Dutch residents (Gijsberts et al., 2012). This suggests that immigrants living in ethnically concentrated neighbourhoods are more likely to lack the social capital which would enable them to succeed in the educational system. Or in a worse case, they might have higher rates of negative social capital, actually working against their positive development (Labianca and Brass, 2006; Nieuwenhuis et al., 2013). Such neighbourhoods will provide adolescents with less knowledge about the educational system and with fewer positive role models, showing them the benefits of education for upward social mobility (Ainsworth, 2002). This may lead to negative attitudes towards education, because the importance of education for upward social mobility is not recognised (MacLeod, 1987).

Looking at it from another perspective, higher concentrations of immigrants might also be beneficial for migrant youth, when shared positive attitudes towards education are combined with a strong co-ethnic social network. First generation immigrants are a group that abandoned their home country to build a life in a new country. For this group, migration is likely to be a strong incentive to perform well in the host country and make use of the available opportunities (Pásztor, 2010). However, many first generation immigrants experience difficulties in overcoming their disadvantaged situation. When immigrants experience difficulties in their own lives to get ahead, they may focus on stimulating the educational success of their children, that way trying to achieve intergenerational social mobility (Zhou and Bankston, 1998). In neighbourhoods with higher shares of immigrants, the likelihood to meet co-ethnics increases. This can give rise to ethnic social networks and institutions, which may help solve education-specific problems and share information; reinforce common norms about education; and offer help in monitoring each other’s children (Portes and MacLeod, 1999; Zhou, 2009). Combining the arguments above would mean that in neighbourhoods with higher proportions of immigrants, the likelihood is higher for the presence of strong ethnic social networks which share the norm that stimulating their children to succeed in the host country’s educational system is important. And, because of more social closure, adult residents are better able to enforce these norms, leading to a higher likelihood for migrant youth to internalise these norms.

Because two opposing ideas exist about how the neighbourhood’s immigrant concentration affects its migrant youth’s educational commitments, we can derive two opposing hypotheses: The higher the proportion of non-Western immigrants in the neighbourhood, the lower the educational commitments of migrant youth (H1a). And: The higher the proportion of non-Western immigrants in the neighbourhood, the higher the educational commitments of migrant youth (H1b). We will examine whether either of the two mechanisms is supported by our results.

For native youth we do not expect to find the same result when studying the effect of the neighbourhood’s immigrant concentration on their educational commitments. Our argumentation for migrant youth depends on them being a minority group in their neighbourhood, and being affected by other migrants due to role models, social capital and ethnic social networks. For natives, however, ethnically mixed neighbourhoods provide a culturally more complex environment. It is not evident that this will affect their educational commitment either positively or negatively.

### Resilient personalities

In the introduction we argued that personality might be a commonly unobserved personal trait that can affect the relationship between neighbourhood characteristics and educational commitment. Previous studies already suggested that there is a relationship between neighbourhood effects and personality traits. For example, studies have found different effects of impulsivity on delinquency between neighbourhoods scoring high and low on indicators for disadvantage (Lynam et al., 2000; Meier et al., 2008; Zimmerman, 2010). Furthermore, neighbourhood characteristics have been found to moderate the effects of low self-control on violent victimisation (Gibson, 2012), of hyperactivity, impulsivity and attention difficulties on conduct problems (Zalot et al., 2009) and of thrill and adventure seeking and lack of premeditation on offending (Jones and Lynam, 2009). In studies on the relationship between the neighbourhood and educational outcomes, personality has however not been introduced yet. Besides, studies thus far have relied on personality traits, while we employ a person-centred approach, using personality types. This approach takes into account the within-person configuration of personality traits, which describes the person as a whole, rather than focussing on specific dimensions of personality (Asendorpf, 2002; Magnusson, 1998). Using personality types takes into account that the meaning of personality dimensions depends on the scores on other dimensions, therewith enabling the study of individuals rather than of mere traits (Scholte et al., 2005).

Personality research offers a useful distinction of three personality types that score differently on ego-control and ego-resiliency: resilients, undercontrollers and overcontrollers (Block and Block, 1980). Ego-control is defined as the tendency to contain versus express emotional and motivational impulses, and ego-resiliency as the tendency to respond flexibly versus rigidly to environmental demands (Klimstra et al., 2010; Meeus et al., 2011). Resilients are characterised by medium levels of ego-control and high levels of ego-resiliency. Undercontrollers score low and overcontrollers high on ego-control, however both score low on ego-resiliency (Asendorpf et al., 2001; Caspi, 1998). Resilients are the best adjusted group and are likely to most effectively cope with neighbourhood influences, because they can respond flexibly and adaptively to environmental demands.

### Resilience as moderator of neighbourhood effects

Above, we argued that a higher concentration of immigrants in the neighbourhood might have either a negative or a positive impact on the development of migrant youth’s educational commitments. When living in a certain neighbourhood exerts an influence on someone’s educational commitments, such neighbourhoods can be described as demanding environments. Because adolescents with a resilient personality are better able to respond flexibly to environmental demands, we hypothesise that the influence of neighbourhoods’ immigrant concentrations on educational commitments is weaker for resilients than for adolescents with non-resilient personality types (H2).

## Data and methods

### Data

Our individual-level data are drawn from the Conflict and Management of Relationships (Conamore) dataset. The Conamore is a panel dataset consisting of 1313 respondents recruited from 12 high schools in the province of Utrecht, the Netherlands. Respondents received a letter inviting them to the study, explaining the project, the goals and the possibility to decline from participating. Both the parents and adolescents provided informed consent. Because the respondents were solely recruited in Utrecht, the data cannot be considered to be representative for the Netherlands. Future research should show whether these analyses are generalisable to other environments. However, we have no reason to expect that the mechanisms regarding the moderating effect of personality will be especially different in Utrecht compared to other places. Regarding neighbourhood immigrant concentrations, it might be that in cities where segregation indices are higher, the disadvantages of high segregation trump the advantages of co-ethnic social capital. The dataset consists of two cohorts: early-to-middle adolescents (*n* = 923; 70.3%) who were on average 12.4 years of age at the first wave, and middle-to-late adolescents (*n* = 390; 29.7%) with an average age of 16.7 years at the first wave. The first wave was collected in 2001/2002, and waves 1 to 5 were collected with a one-year interval, with the data collection of wave 5 in 2005/2006. The sixth wave from 2009/2010 included an additional Life History Calendar (LHC) with retrospective questions from the age of 12 until the timing of the sixth wave. In waves 1, 2, 3, 4, 5 and 6 the numbers of respondents were 1313, 1313, 1293, 1292, 1275 and 1026, respectively. For the first five waves, sample attrition was very low (1.2% across waves). Attrition for the sixth wave is bigger (20%), because of the larger time gap between waves 5 and 6, compared to the one-year gap between the earlier waves. For our analyses we use wave 1 to 5 and the LHC. After listwise deletion of cases with missing values, our sample consists of 901 respondents, of which 812 are natives and 89 are migrant youth. Migrant youth are defined as respondents who have two foreign born parents, and the group consists mainly of Moroccans, Turks, Surinamese and people from the Dutch Antilles (i.e. non-Western migrants). We restricted the analyses to respondents who have at least two observations in different waves. The final N is 4255 (total observations across waves for the 907 respondents), of which 3849 for natives and 406 for migrant youth. Comparing the cases that were discarded due to missing data with the final sample, we find that in the used sample there are slightly more females (56% vs. 54%), fewer migrants (10% vs. 22%), slightly more children from the lowest and highest educated parents (14% vs. 12% and 32% vs. 31%, respondents) and slightly fewer from parents with an educational background in between the extremes (19% vs. 22%). The attrition of migrant respondents may bias our results. It might be that migrant respondents doing poorly in education in particular dropped out of the sample, possibly positively biasing our outcome variables ‘educational commitment’.

Because the Conamore data is geo-coded, and includes all six-digit postcodes (areas containing, on average, 17 households) where respondents lived from the age of 12 onwards, we are able to enrich the data with neighbourhood characteristics on the postcode-level as provided by Statistics Netherlands (CBS, 2011). Because Dutch high schools recruit students from a large area, the respondents are not clustered in postcode areas. The mean number of respondents per postcode area across waves is 1.13, with a maximum of three respondents in one postcode area for 1.2% of the sample. As a result of this lack of clustering, it is not necessary, or even possible, to use multi-level models with a neighbourhood level.

### Measurements

The dependent variable is identification with educational commitments, which refers to the degree that adolescents identify themselves with, feel certain about and internalise the educational commitments they made (Luyckx et al., 2006). It is measured using the Utrecht-Management of Identity Commitments Scale (U-MICS; Crocetti et al., 2008), which consists of five items to measure the degree to which adolescents derive self-confidence from the education choices they have made, with response categories 1 (completely untrue) to 5 (completely true). The items are (translated from Dutch): ‘My education makes me feel confident about myself’; ‘My education gives me certainty in life’; ‘Because of my education I feel certain about myself’; ‘My education gives certainty for the future’; and ‘Because of my education I can perceive the future optimistically’. We constructed scales for educational commitment for the five waves, which all had high reliability (Cronbach’s *α*s 0.90–0.93). The variable is standardised. Descriptive results for this and other variables can be found in Table 1.

**Table 1.** Descriptive statistics.

	Sample: migrant youth (N = 406)				Sample: natives (N = 3849)			
	Mean	S.D.	Min.	Max.	Mean	S.D.	Min.	Max.
*Dependent variable*								
Education commitment	0.16	1.17	−3.75	1.66	−0.02	0.96	−3.75	1.66
*Neighbourhood characteristics*								
Proportion non-Western immigrants: < 0.10	0.14	0.35	0.00	1.00	0.76	0.43	0.00	1.00
0.10–0.20	0.11	0.31	0.00	1.00	0.14	0.34	0.00	1.00
> 0.20	0.75	0.43	0.00	1.00	0.10	0.31	0.00	1.00
*Time-varying covariates*								
Delinquency	0.15	0.29	0.00	1.63	0.15	0.31	0.00	3.00
Family structure (1 = not with both parents)	0.25	0.44	0.00	1.00	0.20	0.40	0.00	1.00
Conflict frequency with parents	0.70	0.51	0.00	2.41	0.65	0.49	0.00	3.08
Parental support	2.52	0.67	0.00	4.00	2.50	0.58	0.04	4.00
Parental power	1.64	0.72	0.00	3.83	1.41	0.61	0.00	4.00
*Time-invariant covariates*								
Resilient personality	0.30	0.46	0.00	1.00	0.45	0.50	0.00	1.00
Gender (1 = female)	0.58	0.49	0.00	1.00	0.56	0.50	0.00	1.00
Parental education: Lower vocational education or less	0.32	0.47	0.00	1.00	0.12	0.33	0.00	1.00
Preparatory middle-level vocational education	0.27	0.45	0.00	1.00	0.19	0.39	0.00	1.00
Middle-level vocational education	0.22	0.41	0.00	1.00	0.19	0.39	0.00	1.00
Higher general continued education or preparatory scientific education	0.17	0.38	0.00	1.00	0.22	0.41	0.00	1.00
Higher vocational education	0.11	0.32	0.00	1.00	0.21	0.41	0.00	1.00
Scientific education	0.15	0.36	0.00	1.00	0.34	0.47	0.00	1.00
Cohort (1 = middle-to-late adolescence)	0.26	0.44	0.00	1.00	0.27	0.45	0.00	1.00

The neighbourhood-level independent variable representing the proportion of non-Western immigrants was measured for 2010. The neighbourhood scale used is the six-digit postcode scale, which pertains to, on average, 17 households. Six-digit postcode areas are a good scale to measure socialisation hypotheses, because socialisation is more likely to happen through neighbours in close proximity than through neighbours living a few blocks away. It was shown that smaller areas more likely represent the individually perceived neighbourhood, and are more suitable to analyse socialisation processes than larger units of analysis (Oberwittler and Wikström, 2009). In a different paper, we were also unable to identify effects for the larger areas (Nieuwenhuis et al., 2015a). When respondents change postcode between waves (which happened at least once for 28% of the sample), this is reflected in different values for the neighbourhood variable in different waves. The proportion of non-Western immigrants is represented by three dummies: < 0.10, 0.10–0.20 and > 0.20,^1^ to allow for the comparison of different degrees of neighbourhood mixing.

**Figure 1.**
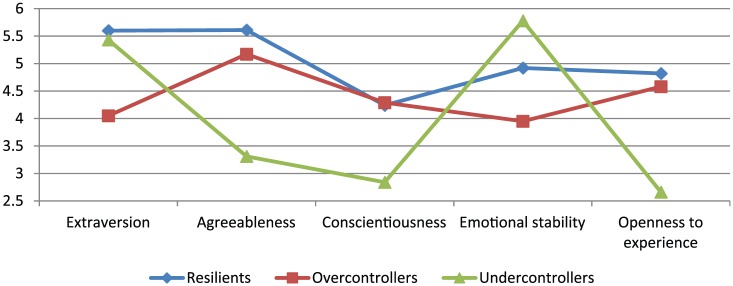
Mean values of Big Five personality traits for the three personality types. *Note*: The scales range between 1 and 7.

From the Big Five personality dimensions we constructed three personality types: resilients, overcontrollers and undercontrollers. The Big Five were measured with a shortened Dutch version of the Big Five questionnaire (Gerris et al., 1998; Goldberg, 1992), containing 30 items, such as: talkative (extraversion), sympathetic (agreeableness), systematic (conscientiousness), worried (emotional stability, reverse coded) and creative (openness to experience). The response categories were 1 (completely true) to 7 (completely untrue). We assessed personality at the first wave in order to have a fixed value for our interaction effects. Cronbach’s *α*s for the Big five scales ranged from 0.77 to 0.87. We used Latent Class Analysis (LCA) to detect latent classes of the most typical configurations of the five personality dimension within persons. The distribution of personality dimensions across different personality types we found corresponds to earlier research (Klimstra et al., 2010; Robins et al., 1996). Resilients score high on all five personality dimensions, and highest on extraversion, agreeableness and openness to experience. Overcontrollers score highest on conscientiousness, but lowest on extraversion and emotional stability. Undercontrollers score highest on emotional stability, but lowest on agreeableness, conscientiousness and openness to experience (see Figure 1). Personality types were stable over the five waves of data for 73.5% of the sample (Meeus et al., 2011). Our interest lies in resilients’ high levels of ego-resiliency versus the low levels of overcontrollers and undercontrollers, so we collapsed overcontrollers and undercontrollers into one category, and created a dummy measuring a resilient personality (1; n = 385) or a non-resilient personality (0; n = 510).

We control for the time-invariant variables gender, parental education and cohort. Furthermore, we control for the time-varying variables: age, delinquency, family structure, conflict frequency with parents, parental support and parental power. Gender is coded as male (0) and female (1). Parental education is measured in a set of six dummy variables, including: 1) lower vocational education or lower (corresponds to ISCED 2011 categories 1 or 2); 2) preparatory middle-level vocational education (ISCED: 2); 3) middle-level vocational education (ISCED: 3); 4) higher general continued education or preparatory scientific education (ISCED: 3); 5) higher vocational education (ISCED: 6); and 6) scientific education (ISCED: 6 or higher). Cohort is a dummy variable coded early-to-middle (0) and middle-to-late adolescence (1).

We control for the time-varying variable delinquency, because delinquency was found to positively correlate with negative school attitudes (Kulka et al., 1982). Delinquency is measured with 16 items about how often the respondent was involved in certain types of delinquent behaviour in the past 12 months, with the following answering categories: 1 (never), 2 (once), 3 (two-three times), 4 (four times or more). Example items are: stole a bicycle, used marihuana or hash, carried a weapon and arrested by the police. We constructed scales for all five waves, with Cronbach’s *α*s 0.82–0.90.

We include several control variables relating to the family, because the parental home is an important context for adolescent development. Parents can support the development of their children’s educational attitudes by being available, by being involved in school and by showing interest in school-related activities (Astone and McLanahan, 1991; Clark, 1983; Coleman, 1988; McNeal, 1999). Also they can exert stricter control over the adolescents’ activities, therewith trying to restrict the negative influence of deviant peers on the development of educational attitudes (Furstenberg et al., 1999; Jarrett, 1997). Parents who have higher frequencies of conflict with their child may be less successful in transmitting their educational aspirations onto their child. Furthermore, adolescents from families with a non-traditional family structure may experience different levels of conflict, control and support. Unfortunately, additional family-related information such as household income and homeownership was not available.

Family structure is a dummy measuring whether the respondent was not living with both parents (1), for every wave. This includes: living with one parent; living with a parent and a stepparent; living alone; or a different situation.

Conflict frequency with parents is measured using a Dutch version of the Interpersonal Conflict Questionnaire, which was reported to have adequate validity (Laursen, 1993). The questionnaire consists of 35 items of potential topics of conflict with five answering categories: 1 (never) to 5 (often). The adolescents reported separately for their father and mother whether they had conflict about topics such as dates, privacy, behaviour in school and homework. The Cronbach’s *α*s range from 0.92 to 0.95 across waves. We combined the scales for fathers and mothers in one scale measuring conflict with parents.

Parental support and power are both measured using the Network of Relationship Inventory (NRI) (Furman and Buhrmester, 1985). The NRI has reported adequate validity (Edens et al., 1999). The questions are asked separately about the father and the mother and use answering categories that range from 1 (little or not at all) to 5 (more is not possible). The support scale consists of 12 items from different subscales of the NRI, such as companionship, instrumental aid, intimacy, nurturance, affection, admiration and reliable alliance. Examples of items are: ‘Do you share secrets or personal feelings with your father/mother?’ and ‘Does your father/mother appreciate the things you do?’. Across waves, the Cronbach’s *α*s range from 0.87 to 0.92. We combined the scales for fathers and mothers in one scale in order to obtain an overall measure of parental support. The power scale consists of six items. Examples items are: ‘How often does your father/mother tell you what to do?’ and ‘To what extent is your father/mother the boss in your relationship?’. Low levels on this scale indicate that adolescents perceive the relationship with their father/mother as equally powerful, high scores indicate that adolescents perceive their father/mother as more powerful. The Cronbach’s *α*s range from 0.81 to 0.90 across waves. Again, we combined the scales about fathers and mothers to obtain an overall measurement for parental power.

## Method

Because we have a panel dataset with five observations over five years for all variables, we are able to employ a hybrid random-effects model, also called a between-within (BW) model.^2^ The BW model is called a hybrid model, because it combines the advantages of both fixed- and random-effects models. It can be written as:

$$y_{ij}=\beta_{0}+\beta_{1}(x_{ij}-{\bar{x}}_{j})+\beta_{4}{\bar{x}}_{j}+\beta_{2}z_{j}+(u_{j}+e_{ij}),$$

where *β*_1_ is the within effect and *β*_4_ is the between effect of a series of time-variant variables *x_ij_* (Bell and Jones, 2015). As fixed-effects models, the BW method estimates the effects of within-person change in the independent variables on the within-person change in the dependent variable. Any time-invariant characteristics (both observed and unobserved) are automatically controlled for, as the sum of their change will always be zero. Therefore, the estimations for time-varying variables in BW models are identical to the estimations in fixed-effects models (Allison, 2009). This removes potential selection bias emerging from time-invariant characteristics that influence both neighbourhood selection and educational commitments (Galster, 2008). Additionally, a BW model includes random-effects and allows for the inclusion of time-invariant variables (*β*_2_), providing additional information on differences between individuals that would not be available in fixed-effects models (for a more detailed description of the method, see Bell and Jones, 2015; Allison, 2009). We ran a Hausman test to examine whether a between-within model is favoured over a random effects model. The result is that the unique errors are likely correlated with the independent variables, making a between-within model the preferred choice. In the tables we report robust standard errors that are clustered on the school from which the respondents were recruited for the first wave of data collection. This is important, because we do not have information on school-level characteristics, however, besides neighbourhoods, schools are considered important contexts for adolescent development, and need to be dealt with. By clustering the analyses on the school level, we take into account some of the school context.

To emphasise the importance of controlling for selection bias, we also employ traditional OLS models and compare the OLS results with the outcomes of the between-within models. OLS models differ from BW models in the sense that OLS estimates are not automatically controlled for all time-invariant characteristics, leaving room for selection bias. Because within-person observations over time are not independent, we cluster the observations on individuals (also in the BW models), thus obtaining more robust standard errors.

We will look at differences between native adolescents and migrant youth (both parents foreign born) by analysing them separately. Next, we will assess our hypothesis that adolescents with a resilient personality experience less influence of the neighbourhood by including an interaction effect between ‘proportion of non-Western immigrants’ and the variable measuring whether or not the respondent had a resilient personality at the time of wave 1.

## Results

To test our hypotheses, we conduct analyses for migrant youth, as well as for native adolescents as a comparative sample (Table 2). Comparing migrant youth (Model 1) and native adolescents (Model 3) we immediately find that natives are not influenced by changes in the neighbourhood proportion of non-Western immigrants, while migrant youth clearly are. We can see that moving from neighbourhoods with less than 10% non-Western immigrants to neighbourhoods with more than 20% non-Western immigrants is related to an increase in the educational commitments of migrant youth. However, moving to neighbourhoods with 10–20% non-Western immigrants has the strongest positive relation with educational commitments. This finding supports the idea that ethnic social capital in neighbourhoods stimulates migrant youth to have stronger educational commitments. Comparing the magnitudes of 0.10–0.20 and > 0.20 with a *χ* ^2^-test, we find that the difference is insignificant (*χ* ^2^(1) = 1.18; p = 0.2782).

**Table 2.** Between-within models on educational commitments: Comparison of natives and migrant youth.

	M1: sample: migrant youth	M2: M1+interaction	M3: sample: natives	M4: M3+interaction
	coef. (s.e.)	coef. (s.e.)	coef. (s.e.)	coef. (s.e.)
*Within-individual*				
Prop. non-West. immigrants (ref.: < 0.10)				
0.10–0.20	1.242 (0.265)**	2.095 (0.413)**	−0.099 (0.062)	−0.046 (0.149)
>0.20	0.969 (0.073)**	1.773 (0.162)**	−0.108 (0.083)	0.039 (0.154)
Prop. n.-W. im. 0.10–0.20*resilient pers.		−1.424 (0.486)**		−0.112 (0.234)
Prop. n.-W. im. > 0.20*resilient pers.		−1.336 (0.191)**		−0.315 (0.197)
Delinquency	−0.295 (0.229)	−0.303 (0.232)	0.016 (0.044)	0.015 (0.044)
Family structure (not with both parents)	−0.077 (0.179)	−0.073 (0.178)	0.008 (0.076)	0.010 (0.077)
Conflict frequency with parents	−0.289 (0.133)*	−0.286 (0.133)*	−0.069 (0.048)	−0.070 (0.048)
Parental support	0.291 (0.108)**	0.282 (0.108)**	0.312 (0.035)**	0.312 (0.035)**
Parental power	−0.019 (0.099)	−0.022 (0.100)	−0.189 (0.044)**	−0.190 (0.044)**
*Between-individual*				
Prop. non-West. immigrants (ref.: < 0.10)				
0.10–0.20	0.153 (0.345)	0.168 (0.700)	0.047 (0.066)	0.111 (0.116)
>0.20	0.670 (0.260)**	0.706 (0.252)**	0.186 (0.074)*	0.265 (0.084)**
Prop. n.-W. im. 0.10–0.20*resilient pers.		−0.056 (0.943)		−0.149 (0.141)
Prop. n.-W. im. > 0.20*resilient pers.		−0.113 (0.484)		−0.192 (0.155)
Delinquency	−0.531 (0.384)	−0.534 (0.388)	−0.349 (0.086)**	−0.343 (0.084)**
Family structure (not with both parents)	−0.208 (0.182)	−0.203 (0.188)	−0.104 (0.051)*	−0.109 (0.051)*
Conflict frequency with parents	−0.085 (0.097)	−0.099 (0.116)	−0.183 (0.064)**	−0.189 (0.066)**
Parental support	0.431 (0.158)**	0.427 (0.152)**	0.378 (0.032)**	0.380 (0.032)**
Parental power	0.198 (0.125)	0.198 (0.121)	0.070 (0.067)	0.072 (0.064)
*Time invariant*				
Resilient personality	0.462 (0.139)**	0.550 (0.529)	0.072 (0.039)†	0.111 (0.049)*
Female	−0.131 (0.207)	−0.136 (0.230)	−0.285 (0.042)**	−0.282 (0.042)**
Parental education (ref.: scientific educ.)				
Lower vocational education or less	0.134 (0.174)	0.132 (0.181)	0.044 (0.097)	0.042 (0.098)
Preparatory middle-level vocat. educ.	−0.309 (0.265)	−0.306 (0.265)	0.001 (0.061)	−0.002 (0.063)
Middle-level vocational education	−0.034 (0.215)	−0.029 (0.219)	−0.023 (0.058)	−0.019 (0.062)
Higher general continued education or preparatory scientific education	−0.389 (0.113)**	−0.395 (0.085)**	−0.118 (0.054)*	−0.112 (0.051)*
Higher vocational education	−0.281 (0.289)	−0.279 (0.268)	−0.031 (0.072)	−0.028 (0.070)
Cohort (ref.: young-to-middle)	0.040 (0.181)	0.033 (0.191)	−0.039 (0.066)	−0.035 (0.064)
Intercept	−1.494 (0.298)**	−1.501 (0.322)**	−0.732 (0.098)**	−0.759 (0.095)**
R^2^	0.2253	0.2268	0.0976	0.0990
N	406	406	3849	3849

*Note*: **p < 0.01; * p < 0.05; † p < 0.10. The values of educational commitment range from −3.75 to 1.66.

To test whether adolescents with a resilient personality are influenced less by the neighbourhood than adolescents with non-resilient personalities, we include interactions in Models 2 and 4 between having a resilient personality and the proportion of non-Western immigrants. For migrant youth, we find that the positive association of higher proportions of non-Western immigrants with educational commitments is still positive, but much weaker for resilients compared to adolescents with non-resilient personalities. We also find that the initial positive relation between being resilient and educational commitments disappears after including the interaction effects. For native adolescents we find no support for such an interaction effect, however we do find a positive relation between having a resilient personality and educational commitments. To find the neighbourhood effect for resilient migrant youth, we run Model 2 with resilients as the reference group. The effect of proportion of non-Western immigrants for 0.10–0.20 is (b = 0.671; s.e. = 0.319; p = 0.035) and for > 0.20 is (b = 0.437; s.e. = 0.076; p = 0.000). The effect magnitude for non-resilient personalities is reflected in the within-individual main effect of proportion of non-Western immigrants (Table 2: M2), which is clearly much stronger than the main effects for resilients. This supports our hypothesis that adolescents with a resilient personality experience weaker neighbourhood effects compared to adolescents with a non-resilient personality type.

Because of the small sample size for migrant youth, we also ran models over the pooled data for both migrant and native adolescents for additional power (results not shown). The interpretation of the three-way interaction between proportion of non-Western immigrants in the neighbourhood, resilient personality and having foreign-born parents leads to the same conclusion as the analyses over separate samples, namely, higher proportions of non-Western immigrants are positively related to educational commitments for migrant youth and not for native adolescents. And for migrant youth, the relation between immigrant proportions and educational commitments is weaker when they have a resilient personality.

As for the controls, the variables on the relationship with parents seem to have logical directions: more conflict is related to less educational commitments (for migrant youth); more parental support is related to more educational commitments; and more parental power is related to less educational commitments (for native adolescents). Delinquency, albeit insignificant, has the expected sign in the migrant youth model. The time-invariant control variables show that native girls have lower educational commitments than native boys. Furthermore, we do not find a strong relation between parental education and educational commitments, and we do not find a relation for the different cohorts.

### Selection bias

Because some studies do find associations between the neighbourhood’s ethnic concentration and educational outcomes, also for native youth samples (for reviews, see: Leventhal and Brooks-Gunn, 2000; Nieuwenhuis and Hooimeijer, 2015), we will examine if we can reproduce these results when not controlling for selection bias. For this purpose, we compare OLS models clustered on individuals, with BW models (Table 3). For migrant youth, the OLS model (Table 3: M1) shows that living in neighbourhoods with more than 20% non-Western immigrants has a positive effect on the educational commitments, compared to living in neighbourhoods with less than 10% non-Western immigrants. Comparing with the BW model (Table 3: M2), we see that the magnitude of the effect of the variable proportion of non-Western immigrants increases, and also the category 10–20% non-Western becomes significant. It is a function of BW models to control for time-invariant unmeasured characteristics that may, in our case, influence both neighbourhood selection and educational commitments. Apparently, for migrant youth, neighbourhood effects become more pronounced when controlling for such characteristics, suggesting that the findings of the OLS models are caused by neglecting to measure certain characteristics.

**Table 3.** OLS vs. BW models on educational commitments: Comparison of neighbourhood characteristics.

	Sample: migrant youth (N = 406)		Sample: natives (N = 3849)	
	M1: OLS	M2: BW	M3: OLS	M4: BW
	coef. (s.e.)	coef. (s.e.)	coef. (s.e.)	coef. (s.e.)
Prop. non-Western immigrants (ref.: < 0.10)				
0.10–0.20	0.351 (0.309)	1.242 (0.265)**	0.019 (0.065)	−0.099 (0.062)
> 0.20	0.761 (0.249)**	0.969 (0.073)**	0.133 (0.080)†	−0.108 (0.083)
R^2^	0.2100	0.2235	0.0872	0.0976

*Notes*: **p < 0.01; * p < 0.05; † p < 0.10. *Note 1*: Both models include the following time-varying control variables: delinquency, family structure, conflict frequency with parents, parental support and parental power. Also, the models include the following time-invariant control variables: resilient personality, gender, parental education and cohort. *Note 2*: The values in the BW models are within-individual coefficients.

When looking at the models for native adolescents (Table 3: M3 and M4), we see that the OLS model suggests a (albeit marginally significant) relationship between the proportion of immigrants and educational commitments, however it vanishes when fitting a BW model. This finding supports the idea that the found neighbourhood effects in the OLS model might actually be family effects, which disappear when controlling for any time-invariant unobserved (family) characteristics. Unobserved characteristics other than those pertaining to the family might also be involved, however it is not possible to distinguish these.

Both comparisons suggest selection bias. For migrant youth, OLS models seem to underestimate neighbourhood effects, while for native youth, OLS models seem to overestimate these effects. This is a strong argument to favour BW models over OLS models, and to some extent this explains the difference between our findings and findings of other studies. It should be noted that the method only controls for time-invariant unobserved individual characteristics, and does not control for time-varying unobserved characteristics, so the presence of selection bias cannot be ruled out totally. However, that we are still able to find neighbourhood effects using this technique adds to the robustness of our findings (also see Nieuwenhuis, 2016).

## Conclusion and discussion

We investigated the effect of neighbourhood immigrant concentration on educational commitments. By employing separate analyses for migrant youth and native adolescents, we find that, as expected, natives are not influenced by this neighbourhood characteristic, while migrant youth are clearly affected by the ethnic composition of the neighbourhood. The results indicate that, for migrant youth, living in neighbourhoods with 10–20% or more than 20% ethnic minorities increases their educational commitments compared to living in neighbourhoods with less than 10% ethnic minorities. This finding brings together the two hypotheses we mentioned: the first hypothesis (H1a) states that there is less native social capital and that there are fewer positive role models in ethnically concentrated neighbourhoods, hindering the development of educational commitments. The other hypothesis (H1b) predicts that ethnic concentration can lead to stronger ethnic social networks, enabling minorities to help each other out, therewith facilitating the development of educational commitments. Our findings suggest that moderate proportions of immigrants in the neighbourhood are most favourable for the development of adolescents’ educational commitments, because in such neighbourhoods they can profit the most from on the one hand contact with natives, bridging the gap to the native society, and on the other hand contact with co-ethnics, giving access to ethnic support networks. However, because we did not specifically test the possible mechanisms behind the neighbourhood effect, more research is needed to disentangle the underlying processes.

We wanted to test whether commonly unobserved characteristics of the research population might alter the relationship between neighbourhood characteristics and individual outcomes. We hypothesised that adolescents with a resilient personality can cope better with environmental stress and demands, and will therefore experience weaker effects of neighbourhood characteristics on their educational commitments than do adolescents without a resilient personality (H2). Our findings show that resilients are indeed influenced less by the neighbourhood’s ethnic concentration. This indicates that resilients are more likely to develop their own value-orientations, despite outside pressures. The support we found for the neighbourhood’s collective socialisation mechanism might thus only work for adolescents who are susceptible to socialisation. This is interesting, because personality theory assumes that resilients are better able to cope with stress and adversity, however it seems that they are also less likely to take in positive environmental influences. Resilients seem to choose their own path amongst alternative commitments.

Our findings shine some interesting light on socialisation mechanisms in neighbourhoods. As mentioned in the introduction, neighbourhood research often takes educational achievement as an outcome, while implying that adolescents are socialised by neighbourhood adults into having certain educational commitments, which consequently influences their achievement. We show that it is indeed quite likely that educational commitments are influenced by neighbourhood characteristics, albeit differently for migrant youth and native adolescents. And other studies have showed that educational commitment is related to greater school performance (Germeijs and Verschueren, 2007; Robbins et al., 2004). It is therefore very plausible that youth’s educational commitments are socialised through neighbourhood characteristics, and consequently influencing their educational achievement. However, other studies have shown that, for migrant youth, educational commitments are not always translated into educational achievement, while for native youth this is more likely to be the case (Elffers and Oort, 2013). It is likely that other factors also influence educational achievement for migrant youth, such as discrimination, stigmatisation or lack of cultural capital, which dissolve the positive influence educational commitments might have. It is likely that the larger part of the differences between native and migrant youth’s educational attainment is explained by other factors than their educational commitments. However, since educational commitments were shown to be related to greater school performance, it is important to know how they come about, in order to be able to stimulate them.

We try to overcome the problem of selection bias by employing between-within models, which control for time-invariant unobserved individual characteristics. In our analyses, we find clear support for the idea of selection bias. However, our respondents, adolescents, are not in a position to choose their own neighbourhood. This decision is made by their parents. This means we are possibly dealing with an intergenerational selection effect (see also van Ham et al., 2014). It is argued that neighbourhood effects are transmittable over generations, i.e. that parents are influenced by their own childhood neighbourhood, shaping their educational and occupational choices and thus influencing their resources later in life, including the resources available for their children and the neighbourhood in which they will raise their children (Sharkey and Elwert, 2011). It is plausible that also neighbourhood selection effects are intergenerationally transmittable: parents on the one hand choose the neighbourhood where they will raise their children and on the other hand influence their children’s educational commitments. Considering these arguments, controlling for selection bias should dissolve any neighbourhood effects. This is true for native adolescents, but for migrant youth we do find neighbourhood effects. Thus, our findings suggest such an intergenerational selection effect for native adolescents, i.e. that parental characteristics that influence neighbourhood choice also influence native adolescents’ educational commitments. For migrant youth, however, controlling for selection actually reveals the neighbourhood effects, suggesting that parental characteristics that influence neighbourhood choice do not similarly influence migrant youths’ educational commitments as well.

When interpreting our results a caveat should be made. We were not able to account for school-level characteristics, other than including the clustering in schools in the calculation of our standard errors. As the demographic composition of neighbourhoods and school may overlap, it is hard to distinguish between neighbourhood and school effects. However, resourceful parents in disadvantaged neighbourhoods may choose to send their child to a better quality school outside their own neighbourhood, leading to social networks outside the neighbourhood, possibly reducing the importance of neighbourhood social networks. Although a meta-analysis of the literature examining the effect of proportions of migrant groups in neighbourhoods on educational outcomes found that including school-level control variables in analyses did not lead to different neighbourhood effects estimators (Nieuwenhuis and Hooimeijer, 2015), for individual studies it might be worthwhile to dig deeper into the complex relation between migrant proportions on schools and neighbourhoods.

In the introduction, we made the point that unobserved heterogeneity might be the reason for the great variation in findings from the neighbourhood effects literature. We introduced two personal characteristics to look into this reasoning: a migration background and a resilient personality. The results clearly show that neighbourhood effects cannot be easily generalised. First, we do not find support for neighbourhood effects on native adolescents, however we do find clear support that migrant youth’s educational commitments are affected by the ethnic composition of the neighbourhood. And second, strong differences seem to exist between adolescents with a resilient personality and those without. The influence of the neighbourhood’s ethnic composition on adolescents without a resilient personality is much stronger than for resilient adolescents. To sum this up, we would like to stress the importance of knowing the background of your research population when examining neighbourhood effects. Failing to account for important individual characteristics could lead to problems in estimating neighbourhood effects. For example, when only a small group is susceptible for an influence of certain neighbourhood characteristics, their effect may be overgeneralised to the whole population of neighbourhood residents, or conversely, the effect may not be found, because it is overshadowed by the large group of residents who are not susceptible. We find significant differences in the effect of neighbourhoods’ migrant concentration on educational commitments between resilient and non-resilient migrant youth. This is a specific case, however, therefore it might be worthwhile to study this process with different outcome variables, different individual traits as moderators and different neighbourhood characteristics.

## Notes

1. We chose these categories, because in the province of Utrecht, and thus in our data, there are not many neighbourhoods with high proportions of non-Western immigrants. Additional categories above 20% of non-Western migrants would result in categories with too few respondents. This is also reflected in the skewedness of the variable, because of which we were not able to include a continuous version of the proportion of non-Western immigrants.

2. We have not looked at lag-effects or first difference models, because the associated reduction in sample size for the model of migrant youth did not leave enough power for such analyses.
